# The Biochemistry of Cytoplasmic Incompatibility Caused by Endosymbiotic Bacteria

**DOI:** 10.3390/genes11080852

**Published:** 2020-07-25

**Authors:** Hongli Chen, Mengwen Zhang, Mark Hochstrasser

**Affiliations:** 1 Department of Molecular Biophysics & Biochemistry, Yale University, New Haven, CT 06511, USA; hongli.chen@yale.edu (H.C.); mengwen.zhang@yale.edu (M.Z.); 2 Department of Chemistry, Yale University, New Haven, CT 06511, USA; 3 Department of Molecular, Cellular, & Developmental Biology, Yale University, New Haven, CT 06511, USA

**Keywords:** cytoplasmic incompatibility, *Wolbachia*, nuclease, deubiquitylase, nuclear transport, histone chaperone, toxin, antitoxin

## Abstract

Many species of arthropods carry maternally inherited bacterial endosymbionts that can influence host sexual reproduction to benefit the bacterium. The most well-known of such reproductive parasites is *Wolbachia pipientis*. *Wolbachia* are obligate intracellular α-proteobacteria found in nearly half of all arthropod species. This success has been attributed in part to their ability to manipulate host reproduction to favor infected females. Cytoplasmic incompatibility (CI), a phenomenon wherein *Wolbachia* infection renders males sterile when they mate with uninfected females, but not infected females (the rescue mating), appears to be the most common. CI provides a reproductive advantage to infected females in the presence of a threshold level of infected males. The molecular mechanisms of CI and other reproductive manipulations, such as male killing, parthenogenesis, and feminization, have remained mysterious for many decades. It had been proposed by Werren more than two decades ago that CI is caused by a *Wolbachia*-mediated sperm modification and that rescue is achieved by a *Wolbachia*-encoded rescue factor in the infected egg. In the past few years, new research has highlighted a set of syntenic *Wolbachia* gene pairs encoding CI-inducing factors (Cifs) as the key players for the induction of CI and its rescue. Within each Cif pair, the protein encoded by the upstream gene is denoted A and the downstream gene B. To date, two types of Cifs have been characterized based on the enzymatic activity identified in the B protein of each protein pair; one type encodes a deubiquitylase (thus named CI-inducing deubiquitylase or *cid*), and a second type encodes a nuclease (named CI-inducing nuclease or *cin*). The CidA and CinA proteins bind tightly and specifically to their respective CidB and CinB partners. In transgenic *Drosophila melanogaster*, the expression of either the Cid or Cin protein pair in the male germline induces CI and the expression of the cognate A protein in females is sufficient for rescue. With the identity of the *Wolbachia* CI induction and rescue factors now known, research in the field has turned to directed studies on the molecular mechanisms of CI, which we review here.

## 1. Introduction

The prevalence of maternally inherited bacterial endosymbionts among invertebrate animals is widely recognized. While mutualists form reciprocally beneficial relationships with their hosts, many symbionts are reproductive parasites that spread by providing reproductive advantages to infected female hosts at the expense of males [1]. *Wolbachia*, a genus of intracellular α-proteobacteria of the order *Rickettsiales*, is the most well-studied of such reproductive manipulators. Since first discovered in *Culex pipiens* mosquitoes nearly a century ago, *Wolbachia pipientis* has been found in many arthropod and filarial nematode species around the world [2,3]. It is estimated that about two-thirds of all insect species carry *Wolbachia* [4]. Since *Wolbachia* are inherited by transmission through the female germline, the reproductive advantage *Wolbachia* provides to an infected female host enhances its own ability to propagate in an insect population [5,6]. Despite being best known as a reproductive manipulator, *Wolbachia* can also be beneficial to certain insect hosts and is an obligate mutualist in filarial nematodes [7,8].

**Figure 1:**
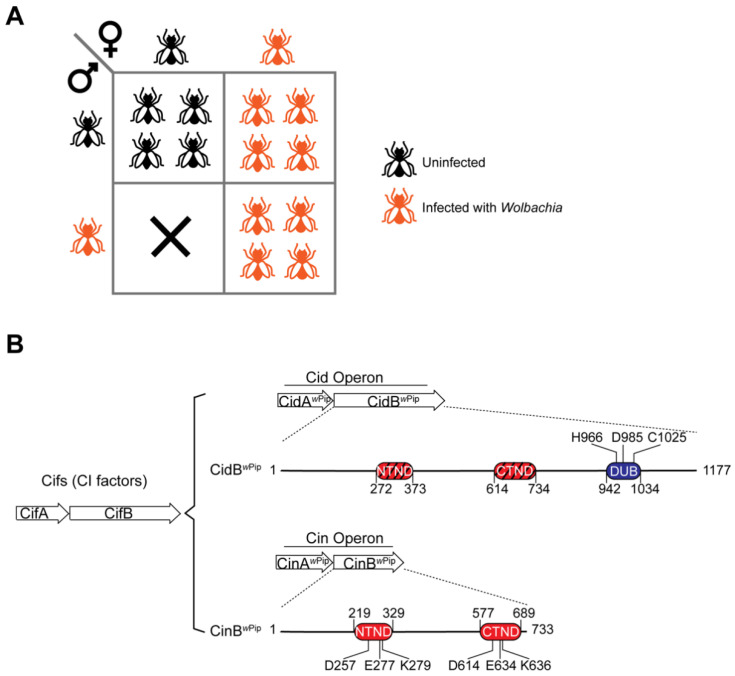
(**A**) *Wolbachia*-induced cytoplasmic incompatibility (CI). Infected females can produce viable offspring with both uninfected and infected males, while uninfected females can only produce viable offspring with uninfected males. Infections are shown in orange. Uninfected insects are shown in black. The figure illustrates so-called unidirectional incompatibility in a diploid host where only infected males mated with uninfected females cause CI, but the reciprocal cross does not. (**B**) Nomenclature of CI factors and examples of *Wolbachia cid* and *cin* operons. In this review, the term Cif is used to generally address all CI factors expressed by the reported *Wolbachia* two-gene operons. The upstream gene within each operon is denoted *A* and the downstream gene *B*. The *cid* operon encodes a deubiquitylase (DUB), CidB, whereas the *cin* operon encodes a nuclease, CinB. The genes in these operons are collectively termed *cifs* for CI factors. CifA refers to the A protein in any CI operon, including CidA and CinA, and CifB refers to the B protein in any CI operon, including CidB and CinB. Both CidB and CinB contain two predicted PD-(D/E)xK nuclease folds. However, the nuclease domains in CidB do not have all the catalytic residues needed for activity (indicated by slashes). NTND: N-terminal nuclease domain. CTND: C-terminal nuclease domain. Numbers shown represent amino acid positions, including catalytic residues within the DUB and PD-(D/E)xK domains.

*Wolbachia*-induced reproductive manipulations include parthenogenesis, the feminization of genetic males, male killing, and cytoplasmic incompatibility (CI) [9]. Among them, CI appears to be the most common in insects [10]. CI is a phenomenon where mating between infected males and uninfected females causes severe developmental defects in the early stages of embryogenesis, which generally results in embryonic lethality in diploid hosts and either gender bias in progeny or embryonic lethality in halploidiploid hosts [11,12,13,14,15]. In diploid hosts, while uninfected females can only produce viable offspring with uninfected males, infected females are capable of producing fully viable progeny with either infected or uninfected males (Figure 1A). Crosses between males and females infected with different *Wolbachia* strains can also be incompatible irrespective of which *Wolbachia* strain is infecting the male or female, a phenomenon termed bidirectional incompatibility. This suppression in infected females of the developmental derangement that would otherwise be caused by mating with an infected male is called “rescue.” Rescue is responsible for the reproductive advantage of *Wolbachia*-infected females in populations where the infection frequency of males exceeds a certain threshold, which depends on factors, such as CI penetrance and the maternal transmission efficiency of the bacteria [5,16].

For decades, *Wolbachia* and CI have been utilized as a strategy to control agricultural pests and reduce the spread of mosquito-borne human diseases [17,18]. Despite the success of using *Wolbachia* in insect population control [19,20,21,22], the molecular mechanisms of CI have long eluded identification. Researchers had proposed a modification-rescue scheme in which CI is caused by a *Wolbachia*-induced modification in sperm from infected males and rescue is accomplished by a factor in the infected egg that reverses or neutralizes the modification [23]. In the past few years, a series of studies has identified several related *Wolbachia* two-gene operons encoding CI factors (Cifs); these appear to be the primary contributors to *Wolbachia*-induced CI and rescue [24,25]. The discovery of these proteins has allowed those studying CI to shift from searching for CI factors to determining how they work. Nevertheless, endosymbionts other than *Wolbachia* can also cause CI, so additional CI factors remain to be discovered [26]. Biochemical analyses of CI mechanisms have only just begun. In this review, we focus on the nature of the *Wolbachia* Cif proteins, their known biochemical activities and genetic effects, and mechanistic models for CI and how they accord with these experimental insights. We also briefly summarize related bacterial effectors that are potentially involved in other reproductive manipulations.

## 2. Cell Biology of Cytoplasmic Incompatibility

CI often manifests itself in the first zygotic division following fertilization. In spermatozoa, the paternal DNA is bound and compacted by small, highly basic proteins, called protamines. Upon fertilization of the egg, the protamines are normally rapidly removed from the DNA and replaced with maternal histones prior to DNA replication [27,28,29]. The female pronucleus migrates towards the male pronucleus until the two abut. Following nuclear envelope breakdown and mitotic chromosome condensation, the two sets of chromosomes align on side-by-side hemi-spindles at the metaphase plate and go through mitotic separation. Only in telophase do the maternal and paternal chromosomes intermingle [30].

The earliest observed embryonic defect from an incompatible cross between a *Wolbachia*-infected male and uninfected female is a delay in the deposition of maternal H3.3 and H4 histones on the genomic DNA of the paternal pronucleus [29]. Protamine removal appears to occur normally. With the delay in paternal nucleosome formation in a CI cross, subsequent paternal DNA replication is prolonged or incomplete. Activation of the cell-cycle kinase Cdk1 and nuclear envelope breakdown in the male pronucleus are then also delayed relative to these events in the juxtaposed female pronucleus [14]. Improper or retarded mitotic condensation of male chromosomes leads to chromosome mis-segregation during anaphase, causing chromatin bridging and aneuploidy that usually leads to the death of the embryo or production of haploid offspring lacking paternal DNA (in the case of haploidiploid hosts) [13,31,32,33].

## 3. The *Wolbachia* CI Factors (Cifs)

The first evidence pointing to the potential CI involvement of what we will call the *cidA–cidB* operon (see below) was a proteomic study that identified peptides derived from the *Wolbachia* CidA protein in the sperm storage organelles (spermathecae) of female *C. pipiens* mosquitoes that had been mated with *w*Pip *Wolbachia*-infected males [24,34]. CidA was not detected in the spermathecae when mating involved uninfected males. While this work did not prove a role for CidA in CI, and the predicted CidB protein from the same operon was not detected in infected sperm, one or more intact *cidA–cidB*-related syntenic gene pairs were observed in the genomes of several other *Wolbachia* strains that cause CI in insects but not in those from filarial nematodes that do not.

Sequence analysis of various CI-inducing *Wolbachia* strains subsequently showed that the *cidA–cidB*-related syntenic genes fall into one of five distinct phylogenetic clades (Types I to V) [25,35,36]. In several examples, these coupled genes are expressed as bicistronic mRNAs [34,37]. Because of the close functional connection in CI between each gene in the syntenic pairs, we will refer to all of them as operons [24,34]. The operons can be further divided into two groups based on the demonstrated enzymatic activity of the B proteins: a *cid* (CI-inducing deubiquitylase) operon that encodes a deubiquitylase (DUB) or a *cin* (CI-inducing nuclease) operon that encodes a nuclease (Figure 1B) [24,38,39]. In this review, we use the nomenclature proposed by Beckmann et al., where the term Cif is used to address all CI factors—that is, CifA refers to any A protein in a CI operon, including CidA and CinA, and CifB refers to the B proteins in CI operons, including CidB and CinB [38]. In the alternative phylogenic classification scheme, Type I Cifs are equivalent to Cids, while types II–IV are all *cin* operon variants. Interestingly, several of the Type V operons encode B proteins predicted to have both active nuclease and DUB domains [36,40].

Studies have since demonstrated that transgenic germline expression of Cifs from either *w*Pip or *w*Mel (a *Wolbachia* strain that naturally infects *Drosophila melanogaster*) in *D. melanogaster* can recapitulate all the key features of CI [24,25]. For example, the transgenic expression of *w*Pip-derived *cidA–cidB* (where *cidB* encodes a deubiquitylase) in the male germline induces almost complete postzygotic male sterility; strikingly, of those embryos observed during the first zygotic division, nearly 90% show the classical CI hallmarks of lagging chromosome condensation in the male pronucleus, anaphase delay, or chromatin bridging [24]. Concurrent experiments with the *w*Mel *cidA* and *cidB* transgenes showed not only strong embryonic defects but also the rescue of these defects by females infected with *w*Mel [25,35,41]. Similarly, the combined expression of transgenic *w*Pip *cinA* and *cinB* genes (where *cinB* encodes a nuclease) in male flies also induces CI-like sterility [39].

Conversely, the germline expression of the cognate *cidA* or *cinA* gene in female flies at least partially rescues these defects [25,35,39,41]. A similar modification-rescue phenotype was observed by the expression of these genes in the budding yeast *Saccharomyces cerevisiae,* where CidB or CinB induces growth inhibition that can be suppressed by the coexpression of the cognate CidA or CinA [24,39,40]. It was the yeast work which first indicated that CidA and CinA would be the CI rescue factors. Crucially, each CidA/CinA protein binds tightly and specifically to its cognate CidB/CinB partner, and this correlates with the strong inhibition of the growth defects caused by the CidB/CinB proteins when they are expressed in yeast [24,39,40].

Sequence analysis of the *cid* and *cin* operons present in different *w*Pip *Wolbachia* strains that naturally infect geographically diverse *Culex* mosquito populations corroborated the above molecular genetic findings. These *w*Pip strains all carry both *cid* and *cin* operons. Strikingly, extensive sequence variation in the *cidA–cidB* operon correlates with bidirectional crossing-type diversity in *w*Pip-induced CI. Variation in the “modification” function of CI is specifically linked to *cidB*, which encodes a DUB [42,43]. The relevance of the *cin*-type operons to CI caused by *Wolbachia* strains, such as *w*No and *w*Yak, on the other hand, was emphasized in recent genomic analyses of these strains, which infect various *Drosophila* species [44,45]. These studies together establish a strong genetic foundation supporting the essential role of the *cif* genes in *Wolbachia*-induced CI.

Interestingly, in most—but not all—CI-inducing *Wolbachia* strains, the Cifs are found as part of a “eukaryotic association module” (EAM) within a viral prophage [36,46]. The link to these WO prophages may reflect how the EAM genes, including those responsible for CI, were acquired by different *Wolbachia* strains; active WO phage particles can be generated from these prophages, a potential means of horizontal gene transfer and possibly even vertical transmission [47].

## 4. Sequence Motifs and Biochemical Activities of the Cif Proteins

The protein sequences of Cifs have been scoured for clues to their potential physiological functions. The clearest similarities to domains with potential enzymatic roles were found in the CidB/CinB orthologs. Type I CifB proteins are the CidB proteases, as noted above (Figure 1B). Their defining Ulp1-like protease domain is responsible for their deubiquitylase activity [24]. Some Type V CifB proteins also include a predicted Ulp1-like domain [36]; none of the CinB proteins (found in Type II–IV Cifs) do. CifB proteins from all five phylogenetic types include two domains with homology to the widespread PD-(D/E)xK superfamily of nucleases [36,48]. Importantly, however, neither of these domains in the CidB (Type I CifB) proteins includes all the residues expected to be essential for catalytic activity, and indeed, no nuclease activity has been detected in *w*Pip CidB [39]. Several Type V Cif proteins are predicted to have both nuclease and DUB activities [24,36,38], although neither activity has yet been demonstrated experimentally in any of them. These are the only motifs identified that are common among the CidB/CinB proteins, particularly those known to contribute to CI. Interestingly, the Ulp1-like domain has been encountered frequently in endosymbiotic bacteria, and proteins with the dual-PD-(D/E)xK nuclease domains, plus a Ulp1-like domain, have been suggested as an ancestral toxin form co-opted in various types of bacterial reproductive parasitism [49].

Based on current data, no intrinsic enzymatic activity is likely for any of the CidA/CinA proteins, although several (weak) sequence similarities to known protein domains have been pointed out [35,36,37]. A short sequence element (21–22 residues long) that is related at low probability to catalase occurs specifically in CidA (Type I CifA) proteins. Catalase is a large tetrameric enzyme that requires a heme cofactor for its ability to decompose hydrogen peroxide into water and oxygen. Therefore, the short “catalase-rel” segment in CidA is not likely to have this activity. Second, a segment near the C-terminal end of CifA has been suggested to have homology to STE transcription factors in the Type I-IV CifA proteins. It would be intriguing if a DNA-binding activity could be traced to the CifA proteins, but the prediction is not of high confidence [37]. Finally, weak similarity to Puf family RNA-binding domains has been reported in all five CifA types [36,37]. The Puf domain comprises a series of helical repeats similar to Armadillo (Arm) and HEAT repeats, which are well known protein-binding motifs. It will be of interest to determine if the putative Puf-like domain in CifA proteins binds RNA or protein and if such binding is relevant to CI.

An attempt to test the potential physiological significance of these motifs by mutagenesis of the *w*Mel *cidA* and *cidB* genes and analysis of the mutants in transgenic fruit flies was recently described in a preprint by Shropshire et al. [50]. Mutation of the critical DUB active-site cysteine to alanine in CidB*^w^*^Mel^ abolishes CI when coexpressed with CidA*^w^*^Mel^ in male flies, confirming the effect previously seen when the same mutation was made in CidB*^w^*^Pip^ [24]. Notably, this CidB*^w^*^Pip^ mutant had been tested in yeast where its expression level was similar to that of the wild-type enzyme, and the mutation did not alter the protein’s ability to bind CidA [24]. In addition, structural studies of Ulp1-related proteins show that modification of the solvent-exposed catalytic cysteine does not lead to significant changes in protein folding [51].

The disruption of either of the (catalytically inactive) PD-(D/E)xK motifs in CidB*^w^*^Mel^ prevented transgenic CI [50], consistent with these pseudonuclease folds having either structural roles or functions in nucleic acid binding. Similarly, CidA*^w^*^Mel^ alleles with multiple mutations disrupting the unannotated N-terminal region, the catalase-rel element, or the Puf-like domain impaired CI induction, whereas the STE domain mutant functioned normally. Potentially the most interesting mutant was the Puf domain disruption as far as the mutant strongly impaired CI induction but not its rescue function. These results are provocative, but one must be cautious in interpreting them without knowing if the folding or expression levels of the proteins were altered. In all the alleles studied by Shropshire et al., other than the catalytic CidB mutant, multiple mutations were made in conserved residues and involved the alteration of at least one bulky hydrophobic residue. For example, the CidA*^w^*^Mel^ Puf domain mutant had four mutations (L287A, S288A, L348A, I349A). Even the apparent separation-of-function Puf domain mutations might cause defects only in CI induction and not rescue because of different levels of expression needed for these functions in the transgenic male and female germlines, respectively.

At present, there is direct experimental evidence for three biochemical activities in the Cifs: deubiquitylase activity in the CidB proteins, DNAse activity in CinB, and high-affinity, cognate-specific binding between CidA–CidB and CinA–CinB protein pairs [24,39]. CidB*^w^*^Pip^ has also been shown to bind, either directly or indirectly, to a number of host factors [40]. It is possible that additional biochemical activities that modulate CI will be found in the Cif proteins, such as DNA or RNA binding [24,37,39]. In the following sections, we review what is known about the biochemically distinct CidA–CidB and CinA–CinB factors and explore how they might account for CI induction and rescue caused by *Wolbachia* infection.

## 5. CidA–CidB, a CI Inducing Protein Pair Containing Deubiquitylase Activity

**Figure 2:**
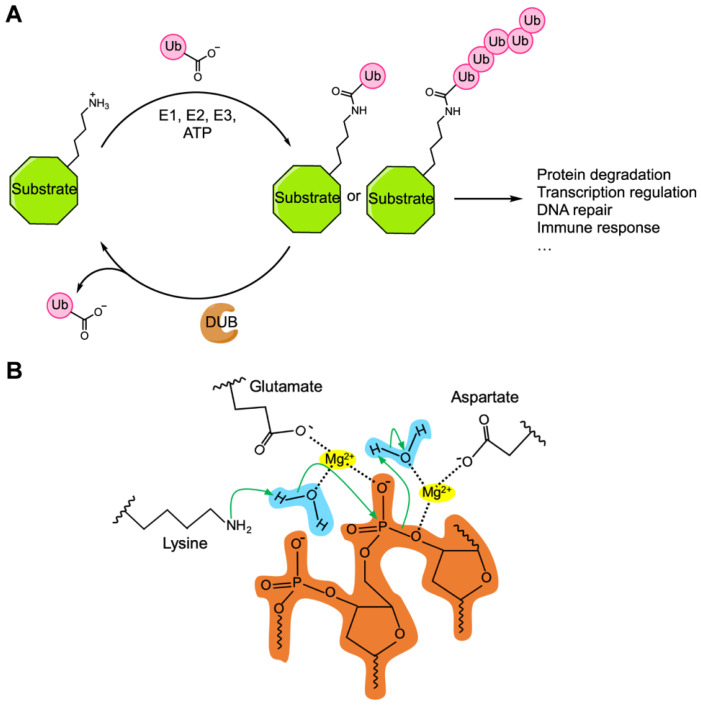
(**A**) Summary of the ubiquitin system. In eukaryotes, the small protein modifier ubiquitin (Ub) can be covalently attached to a nucleophilic residue of its protein substrate by the combined actions of the E1 ubiquitin-activating enzyme, an E2 ubiquitin conjugating enzyme, and an E3 ubiquitin ligase in an ATP-dependent manner. Polyubiquitin chains can form when a ubiquitin is further modified by other ubiquitin molecules. Ubiquitin modification is reversed by the enzymatic activity of deubiquitylases (DUBs). (**B**) Mechanism of PD-(D/E)xK nucleases. Catalytic aspartate and glutamate coordinate two magnesium ions (yellow) that bring two water molecules (blue) near the cleavage site. Catalytic lysine deprotonates and activates one of the water molecules, which then serves as a nucleophile that attacks the DNA phosphate backbone (orange). The second water molecule, along with the two magnesium ions, help stabilize the reaction intermediate. Green arrows represent electron pair movements.

As mentioned above, the first pair of Cif proteins discovered included a protein, CidB (Type I CifB), with deubiquitylase activity [24]. In eukaryotes, ubiquitylation is a common post-translational modification in which the small protein ubiquitin is covalently attached to a nucleophilic residue, typically lysine, in the protein substrate [52] (Figure 2A). Ubiquitin can be further linked with other ubiquitin molecules through one of its seven lysine residues (K6, 11, 27, 29, 33, 48 and 63) or its N-terminal methionine amino group (M1) to form amide-linked polyubiquitin chains [53]. In cells, protein ubiquitylation is highly dynamic, controlled by the opposing actions of ubiquitin-ligating enzymes, which activate and attach ubiquitin to its protein substrates, and DUBs, which remove ubiquitin from the modified proteins or ubiquitin chains [54,55]. The ubiquitin system provides a versatile machinery to regulate many cellular processes, such as protein degradation, transcription, DNA repair and cellular immune responses [56,57,58].

While bacteria do not have a ubiquitin system themselves, a number of intracellular bacteria have been found to secrete effector proteins into the host cytoplasm that alter host ubiquitylation to benefit bacterial survival and propagation [59,60]. Many of these effectors contain DUB domains, which show strong similarity to DUBs or other proteases found in eukaryotes, indicating that they were likely acquired by the bacteria through horizontal gene transfer [59,61]. The majority of the reported bacterial DUB effectors have been found in pathogenic bacteria, such as *Salmonella*, *Chlamydia* and *Legionella* species [59,60], where their DUB activities protect the bacteria from ubiquitin-mediated xenophagy (autophagic elimination of the endocytosed bacteria) and dampen NF-κB-dependent immune responses [62,63]. As noted above, more recent studies have revealed a number of DUB domain-containing effectors that are potentially involved in host reproductive manipulation by bacterial endosymbionts [24,25,49], including CidB from *Wolbachia*.

Near its carboxy-terminus, CidB contains a ~90-residue CE clan/Ulp1-like domain, which defines the CE clan proteases. These most commonly target SUMO (small ubiquitin-like modifier), a ubiquitin-like protein (Ubl) in eukaryotes [24,64,65]. In bacteria, however, the CE clan proteases often behave as DUBs. Intriguingly, several of these proteases are also acetyltransferases [66,67]. Recombinant CidB purified from *Escherichia* *coli* can be covalently modified with an active site-directed ubiquitin suicide probe; it also demonstrates DUB activity against a ubiquitin-AMC fluorogenic substrate as well as polyubiquitin chains. By contrast, it shows no cleavage of SUMO-derived model substrates. It is weakly active against another Ubl model substrate, NEDD8-AMC [24]. This substrate preference resembles some other bacterial proteins with Ulp1-like domains such as RickCE from *Rickettsia bellii* and the recently discovered OtDUB from *Orientia tsutsugamushi* [61,68].

DUB substrate specificity can also give clues about function [60]. When tested with a panel of di-ubiquitin molecules linked through different lysines, CidB is able to hydrolyze all seven different lysine linkages but with a preference towards the K63 linkage [24]. Despite its broad specificity towards different ubiquitin–ubiquitin linkages, CidB expression in yeast does not detectably alter the bulk ubiquitin-conjugate pattern in whole cell lysates, suggesting it has a limited range of substrates in cells [24]. Based on the observed defects in the paternal pronucleus in embryos from incompatible crosses and a previous genetic study that ruled out extranuclear paternal factors as CI targets, the target(s) of the CidB DUB most likely lies in the sperm nucleus or male pronucleus [29,69]. Within the nucleus, K63 ubiquitin-chain modifications are most often associated with genome maintenance, such as recruiting DNA repair proteins to damage sites. Such factors could be CI-relevant CidB substrates [70,71].

In transgenic male fruit flies expressing the *w*Pip *cidA–cidB* operon, the mutation of the CidB active site from cysteine to alanine completely abolishes CI induction, indicating the important role of the DUB activity in CI [24]; the recent transgenic fly study with mutant CidB*^w^*^Mel^ corroborates this [50]. The findings are consistent with CidB-induced growth inhibition in yeast, which is also dependent on its DUB active site [24]. An important difference between the yeast and transgenic fly results is that, to date, the induction of CI-like male sterility by transgenic expression in male flies also appears to require co-expression of CidA. In fact, transgenic expression of CidB*^w^*^Mel^ alone in male flies fails to induce CI, while the same transgenic system induces strong CI when CidA and CidB are expressed together [25,41]. This has raised the question of whether CidB is the exclusive CI-inducing factor from *cidA–cidB* (see below) [25,41].

To understand how CidB contributes to CI, the ubiquitylated substrate(s) targeted by the CidB DUB will need to be identified. A recent report has pointed to proteins involved in nuclear import and histone deposition as processes linked to CidB [40]. Extracts from *D. melanogaster* adults were incubated with a resin coupled to catalytically inactive CidB (hereafter CidB *) as the bait to identify fly proteins that can bind CidB *. Among the top hits were the karyopherins Kap-α2 and Moleskin (a karyopherin β family member), as well as histone chaperones P32 and Nap1 [72]. Notably, an independent screen in yeast for high-copy suppressors of CidB-induced growth inhibition identified the Kap-α2 ortholog Kap60 (Srp1) as the strongest suppressor [40]. The overexpression of P32 or Kap-α2 in the *Drosophila* female germline could partially suppress CI caused by *w*Mel infection of males. Preincubation of the CidB * resin with CidA dramatically alters the interactome of CidB *, including the disruption of its interactions with both karyopherins and histone chaperones. CidA alone, surprisingly, only interacts with a very small number of *Drosophila* proteins, suggesting that its ability to cause rescue and suppress CidB-induced CI may lie principally in its ability to interfere with CidB binding to its critical targets. Alternatively, it may involve interactions with other ligands, such as RNA or DNA.

Whether any of these CidB-interacting proteins are ubiquitylated proteins that are deubiquitylated by CidB remains to be determined. However, these findings are intriguing because of their connection to the earliest known defects observed in embryos from incompatible crosses (see above), which is the delay in maternal histone incorporation into the chromatin of the male pronucleus [29]. In a recent study of the transcriptome of *Tetranychus urticae* (spider mite) embryos, genes involved in histone modifications and the histone genes themselves were among the genes whose expression significantly changed in *Wolbachia*-mediated CI crosses compared to wild-type or rescue crosses [73]. CidB may cause defects in histone deposition by altering the ubiquitylation of histones or histone chaperones involved in nucleosome formation. Alternatively, if the karyopherins identified were the key substrates of CidB, the enzyme may prevent the nuclear import of maternal factors important for protamine–histone exchange in the paternal pronucleus. The interaction between CidB and karyopherins can also be interpreted as a means to localize CidB to the nucleus, where its target(s) is likely located. The stark differences between the interactome of CidB * and the CidA/CidB * complex would be consistent with the hypothesis that CidA rescues CI by preventing CidB from interacting with its substrate(s) or by altering CidB subcellular localization.

While the DUB activity of CidB is necessary for transgenic CI, it is not known whether it is sufficient, and indeed, it is likely that it is not. For example, the CidA–CidB interaction, which does not require the DUB domain, is likely essential for CI rescue and potentially CI induction as well. Hence, regions of the protein necessary for the cognate-specific association of these proteins would also be predicted to be necessary for CI. Domain analysis of various Cifs revealed that they have a highly mosaic nature, suggesting that multiple domains may cooperate to induce CI [49]. Other CE clan bacterial DUB effectors have been shown to contain additional accessory domains that contribute to the biological functions of the effectors. For example, SseL, a *Salmonella* effector protein with an Ulp1-like domain, also has a VHS domain that facilitates its localization to the *Salmonella*-containing vacuoles [61]. Identifying the functions of other putative domains in CidB, such as the elements that mediate CidA binding, will therefore be important to understand the molecular mechanism of Cid-induced CI. CidB copurifies with DNA when expressed in *E. coli* [24]. This may be mediated by the inactive PD-(D/E)xK nuclease domains, which might facilitate CidB association with chromatin, for example, during protamine–histone exchange. The precise roles of the pseudo-PD-(D/E)xK domains in CidB-dependent CI remain to be addressed.

## 6. CinA–CinB, a CI Inducing Protein Pair Containing Nuclease Activity

The other broad biochemical class of *Wolbachia* Cifs that has recently been shown to induce transgenic CI and rescue is the *cinA–cinB* nuclease operon of *w*Pip [39]. The *cin* operon was initially discovered in a *w*Pip *Wolbachia* strain through its protein sequence similarity to the *w*Pip *cid* operon [34]. *w*Pip CinA and CinB share about 50% and 40% sequence similarity, respectively, with the paralogous Cid proteins in the same *Wolbachia* strain [24]. Similar to the *cid* operon, mating between male flies transgenic for *cinA*–*cinB* and uninfected wild-type females induces CI-like embryonic lethality that can be rescued if females are transgenic for *cinA* gene [39]. The expression of *w*Pip *cinB* in yeast also induces a temperature-dependent lethality that can be suppressed by co-expressing *w*Pip *cinA* [24,39]. Furthermore, the nuclease activity of CinB is essential for its ability to induce CI in flies and growth inhibition in yeast since a catalytic lysine-to-alanine mutation inhibits both its nuclease activity and ability to impair growth and development, highlighting the physiological importance of CinB nuclease activity [24,39].

The CinB CI-inducing DNase contains two PD-(D/E)xK nuclease domains (Figure 1B) [24,39,49]. Both nuclease domains share similar predicted structural features, such as the αβββαβ core topology and a trio of catalytic residues—aspartate, glutamate and lysine—that are highly conserved among most PD-(D/E)xK nucleases [39,74,75]. Both of these nuclease domains are predicted to coordinate a divalent cation(s) via conserved aspartate and glutamate residues, while the conserved lysine side chain functions as a general base to activate a water molecule (Figure 2B) [39,76]. Each of the PD-(D/E)xK domains in CinB also possesses a second conserved glutamate residue in the first α-helix of the nuclease fold [39]. These glutamates are known to contribute to active-site formation among some members of the PD-(D/E)xK nuclease superfamily [76,77].

Interestingly, the conserved catalytic residues in both nuclease domains of CinB are required for the protein to maintain its nuclease activity in vitro and ability to induce growth defects in yeast, although the expression of the mutant proteins is not affected [39]. Many PD-(D/E)xK nucleases can form dimers, where DNA cleavage requires both monomers to interact with DNA recognition sites [78]. It is possible that the two nuclease domains in CinB mimic the nuclease dimer and are involved in substrate recognition and DNA cleavage in a coordinated manner. The N-terminal nuclease domain in CinB is potentially the primary DNA recognition domain, based on the observation that a CinB mutant protein with the N-terminal domain carrying a catalytic lysine-to-alanine mutation could no longer bind DNA strongly, while the analogous mutation in the C-terminal catalytic site did not reduce DNA binding (but did eliminate DNase activity) [39].

The observed DNase activity of CinB in vitro is relatively weak and shows broad specificity [39]. Substrates include supercoiled DNA, linear double-stranded DNA, and both single- and double-stranded deoxyoligonucleotides. The weak activity is consistent with the ability to express recombinant CinB in *E. coli* with little impairment of growth. It is likely that the nuclease either has stronger activity against particular DNA sequences or structures or requires activation by a host cell factor. Furthermore, despite lacking activity against tRNA and rRNA, the possibility remains that CinB has nuclease activity against other RNA substrates, such as mRNA. In fact, mRNA is the most frequent cellular target for bacterial toxins in toxin–antitoxin (TA) systems [79]. Eight out of thirty-four TA systems identified in *E. coli* encode toxins that regulate mRNA stability [80]. These toxins, collectively termed mRNA interferases, can target host cellular mRNA in either a ribosome-independent manner [81], where the toxin cleaves specific mRNA sequences by itself, or a ribosome-dependent manner [82,83], where the toxin can only cleave mRNA when it associates with the ribosome [79].

It has been proposed that the Cifs target molecules or biological machineries involved in mitosis that are conserved among insect species because similar CI-induced cytological defects, such as chromatin bridging and early embryonic mitotic arrest, have been observed across a wide range of insect hosts [9,24,84]. Additionally, the successful establishment of heterologous *Wolbachia* infections in diverse insect hosts by embryonic microinjection still usually results in the induction of CI [16,85,86,87,88]. Based on the fact that CinB is a nuclease and CI causes paternal chromosomal defects [69], the most intuitive hypothesis for how CinB induces CI is that the nuclease targets DNA from the sperm, causing DNA damage that leads to incomplete or defective paternal DNA replication following fertilization. The delays in histone deposition on paternal DNA, paternal nuclear envelope breakdown, and activation of the mitotic kinase Cdk1, as well as abnormal paternal chromosome condensation and segregation have all been speculated to be downstream effects related to a fundamental DNA defect in the paternal chromosome set [13,14,29,33]. In the previously mentioned transcriptome analysis of *T. urticae* embryos from CI crosses, the DNA damage response was among the upregulated gene pathways [73].

DNA damage can activate cell-cycle checkpoints that pause cell-cycle progression and allow time for DNA repair, but the checkpoint may not be fully active in embryos [30,89]. Many bacterial nuclease toxins have been shown to induce host DNA damage responses and cause growth arrest and DNA degradation [90,91,92]. In insects, the paternal and maternal chromosomes reside in distinct regions of the metaphase plate separated by a partially broken-down nuclear envelope during the first mitotic division following fertilization [30,32]. While maternal chromosomes proceed normally through mitosis, the paternal chromosomes are forced to enter mitosis with improperly replicated DNA, resulting in abnormal chromosome condensation and segregation [32]. Even though the *cid* and *cin* operons are likely to have different biochemical mechanisms of CI induction, they both induce very similar embryonic cytologic defects. This suggests the two operons might target similar biological pathways [25,39,93,94]. For instance, it is possible the two PD-(D/E)xK nuclease domains, even if catalytically inactive, facilitate the localization of CidB to chromosomal DNA where it can inactivate targets, such as proteins involved in DNA repair or chromatin assembly.

## 7. Models of CI Induction and Rescue

Many ideas have been suggested for the mechanism of CI (Table 1). Most fall under the general formalism of a modification-rescue scheme, as outlined by Werren [23]. A modification function from *Wolbachia* is proposed to modify the sperm during spermatogenesis—since no *Wolbachia* remain in mature sperm—and upon fertilization. The infected egg provides a rescue activity that reverses or neutralizes the original sperm modification.

One of the earliest models that fits under the modification-rescue rubric is the mis-timing model (also known as the slow-motion or pronuclear timing model), which proposes that *Wolbachia* infection delays male pronuclear mitotic progression relative to the female pronucleus during the first zygotic division and that rescue is achieved by either similarly slowing down the development of the maternal pronucleus to compensate for this delay or accelerating the paternal pronuclear development to reach developmental synchronization [14,31,32,95]. This model was initially proposed based on the observed cytological phenomenon of CI where the paternal chromosomes mis-segregate despite proper mitosis in the maternal pronucleus [31]. Evidence supporting this model includes the findings that CI delays nuclear envelope breakdown and Cdk1 activation in the male pronucleus relative to the female pronucleus and that rescue restores cell-cycle synchrony [14]. Furthermore, in mammalian cells, the fusion of cells at different points in the cell cycle can cause chromatin condensation abnormalities reminiscent of CI [96]. If CI defects were a result of asynchrony between the paternal and maternal pronucleus, as some variations of the mis-timing model state, it would indicate that the *Wolbachia*-modified paternal pronucleus remains functional and, if given enough time, should remain capable of normal mitotic divisions and thus support androgenetic embryonic development—i.e., production of adult progeny solely from the paternal pronucleus. However, it has been shown that *Wolbachia* infection inhibits such development, suggesting that CI may involve defects additional to the observed temporal delay in paternal pronuclear development. Alternatively, asynchrony between the paternal pronucleus and certain cytoplasmic cell-cycle components independent of the maternal pronucleus could be what induces CI [97]. The Cif factors could be responsible for this latter asynchrony by inducing replication and mitotic delays in the male pronucleus.

Another early model is the titration–restitution (“sink”) model. In infected males, *Wolbachia* are proposed to remove a key host factor from the paternal DNA during spermiogenesis. Upon fertilization, the deficit of this factor in the male pronucleus delays male pronuclear progression to mitotic division. If the female is infected with a compatible *Wolbachia* strain, the chromatin factor is comparably depleted from the maternal DNA but the *Wolbachia* in the egg eventually restore the factor equally to both pronuclei [95,98]. This model was originally proposed based on the finding that a series of monoclonal antibodies developed using purified *Wolbachia* as the immunogen can recognize a number of host proteins including histone H1, suggesting the binding of *Wolbachia* to these host proteins [98].

A third model, called “lock and key”, suggests that *Wolbachia* deposit a “lock” factor during spermatogenesis that binds to a certain component(s) of the paternal nucleus and interrupts its normal function. To rescue CI, *Wolbachia*-infected eggs produce a “key” to physically interact with the “lock” and remove it from the paternal material [23,99,100,101]. The “lock and key” model distinguishes itself from the other two models by emphasizing that the induction and rescue phenomena are achieved by two different *Wolbachia* factors, and rescue requires physical interaction between the induction and rescue factors.

Since the discovery of the Cifs as the key mediators of CI, two distinct models, inspired by the early models, have been advocated by different groups [38,41]. One is the toxin-antidote (TA) model (which closely resembles the “lock and key” model), which proposes that a “toxin” molecule induces CI by altering some aspect of the sperm nucleus or paternal pronucleus and a second factor, the “antidote” or “antitoxin”, provisioned in the *Wolbachia*-infected egg is responsible for rescue by direct binding to the toxin [24,38,100]. One important point to note about this terminology is that the toxin need not be lethal to host cells such as sperm or fertilized embryos, but rather accounts for inducing some abnormality, especially in the paternal pronucleus. The other model is the “two-by-one” model, which in its simplest form just notes that two *Wolbachia* factors are necessary for inducing CI, while only one of these two factors is required for rescue [41]. The two-by-one scheme was later elaborated such that it could accommodate both TA and host-modification (HM) models [102]. The latter emphasizes the pre-fertilization modification of sperm by the Cifs and post-fertilization reversal of the modification by CifA. In the HM models, Cifs induce and rescue CI either by tuning the relative timing of male and female pronuclear development (as is proposed in the mis-timing model) or modify and restore an important testis-specific host factor(s). In all of these models, CifA is the rescue factor. Below we will discuss these and the other earlier models to see how well they comport with the biochemical and genetic data available on the Cif proteins.

All existing models can be reconciled to some degree with our current knowledge of the Cifs. Without further insight into the exact host targets and sites of activity of the Cifs, their general involvement in delaying/accelerating relative pronuclear development or binding and modifying important host factors is plausible. The lock-and-key model accords with the strong protein–protein interaction between cognate CifA and CifB pairs. In the mis-timing and sink models, on the other hand, CifA and CifB together may function as a complex in the sperm to induce the respective host modification while CifA alone somehow acts as the rescue factor to reverse this. An alternative explanation, as in a more recent version of the two-by-one scheme, is that CifA functions as the major CI-inducing protein as well as the rescue factor, with CifB only having an “adjunct” function in CI induction [50]. Intriguingly, the proposal of the sink model was partly based on the observation that *Wolbachia* likely interact with histones. The evidence that CinB is a DNase and that CidB physically interacts with histone chaperones is consistent with this observation.

The TA and HM models focus on explaining the CI mechanism via the actions of CifA and CifB. The HM model [102] differs from the TA model primarily in three areas: (1) which Cif is the primary CI modifier; (2) whether CifB is carried into the embryo by sperm; (3) whether CifA rescues the defect by directly reversing the modification or by binding and inhibiting CifB. We have argued, based on the established biochemical and genetic properties of the Cifs, that CifB is a CI-inducing factor and CifA a rescue factor [24,38,39]. As discussed earlier, the *w*Pip CidB and CinB proteins induce growth defects in yeast when expressed alone, but this defect is largely suppressed by the co-expression of the cognate CidA or CinA protein to which it binds. Moreover, in diverse *w*Pip-infected *C. pipiens* mosquito populations, variation in the “modification” function of CI is specifically linked to the diversity of the *cidB* gene [42,43].

One observation that would seem to argue against the TA model is that, in transgenic male flies, the co-expression of CifA and CifB is necessary to strongly decrease embryo hatch rates, unlike in yeast where CifB alone induces growth defects [24,25,39]. This result suggests that CifA and CifB might work together to fulfill the modification/toxin role in CI. We can suggest several possible explanations. During spermiogenesis, *Wolbachia* bacteria are removed to the “waste bag” organelle prior to final sperm maturation [6]. At earlier stages in the developing cysts of the insect testis where *Wolbachia* are abundant, CifB might need to be kept inactive to prevent premature CI-related defects. Tight binding by the cognate CifA antidote would be one way to achieve this. In addition, CifA association might enhance CifB packaging into the mature sperm or its delivery from sperm to egg. In short, CifA might have an accessory role in the CI-inducing function of CifB. These mechanisms would fit the contours of the two-by-one description of the apparently opposite functions of CifA in CI induction and rescue [35,41,102].

The authors of the two-by-one/HM model, by contrast, argue that CI induction is most consistent with CifA as the major inducer because a complex mutation of the putative Puf domain in CifA abrogates CI induction without affecting rescue [50]. If CifA were antagonizing CifB to prevent premature defects in the testes by the same mechanism it uses against CifB for rescue in the embryo (i.e., through direct physical binding), then such a separation-of-function mutation should not be possible. As noted above, there are caveats to these experiments, but even if correct, CifA could have ancillary functions, as just noted, that are required for CI induction but not rescue. Therefore, the TA and two-by-one/HM models can both accommodate these genetic results.

**Figure 3:**
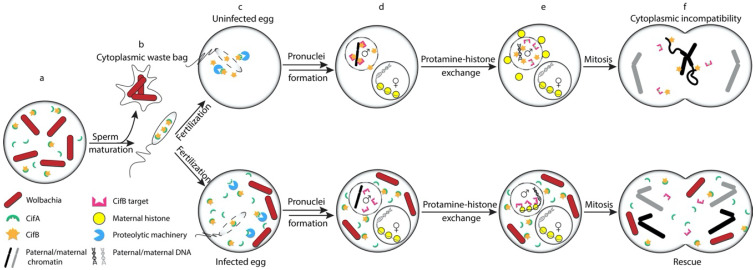
Hypothetical molecular mechanism of CI and rescue by the *Wolbachia* CI factors based on the TA model. a. *Wolbachia* CifA and CifB proteins are secreted into the host testis cells. Under normal conditions, CifA binds tightly to CifB to inhibit its premature action during spermatogenesis and facilitate its delivery to the sperm. b. During spermiogenesis, *Wolbachia* are shed from the mature sperm while CifA and CifB proteins remain in the sperm. c. Upon fertilization, CifA is rapidly degraded by a protease(s). Free CifB protein is now active (Top) unless the egg is infected by the same (or a compatible) strain of *Wolbachia* that can supply the embryo with fresh CifA protein (Bottom). d. (Top) In an uninfected egg, free CifB likely localizes to the paternal pronucleus where it can interact with paternal chromatin and modify its target(s). (Bottom) If the egg is infected with a compatible strain of *Wolbachia*, newly synthesized CifA secreted by the bacteria changes the localization of CifB through direct protein–protein interaction, preventing CifB from accessing its target(s). e. (Top) CifB modification of the target (shown as green star on the target) results in aberrant histone deposition and other downstream defects in the paternal pronucleus. (Bottom) CifB neutralization by CifA results in normal protamine–histone exchange in the paternal pronucleus. f. (Top) Improper condensation of the paternal chromosome leads to chromosome mis-segregation during anaphase with chromatin bridging and shearing of paternal DNA. (Bottom) Properly condensed paternal chromosomes in the “rescued” embryo allows for normal mitotic division.

An important feature of the TA model is that rescue occurs through direct binding of the antidote to the CI modification factor/toxin, preventing it from modifying its CI-relevant target(s) (Figure 3) [95]. As noted above, in vitro binding experiments demonstrate that CifA binds to CifB in a cognate-specific manner [24]. These interactions, at least between CinA and CinB, are very strong (K_d_ ~25 nM) [39]. Thus, a reasonable first hypothesis had been that the rescue effects of CifA observed in transgenic fruit fly studies are achieved by the inhibition of CifB catalytic function through direct protein binding by CifA. However, for both CidB and CinB, cognate CidA or CinA binding does not inhibit their catalytic function in vitro, suggesting that CifA rescues CI by a different mechanism [24,39].

It is possible that CifA association changes the cellular localization of CifB in vivo, a common way of regulating substrate specificity of DUBs [104]. As mentioned above, the CidB protein interacts with both karyopherin α, a nuclear import receptor, and the P32 histone chaperone from *D. melanogaster* protein extracts [40]. These data suggest that CidB could potentially localize to nuclei; CidA binding might hinder CidB from entering nuclei and accessing its target substrates. Similarly, CinA might limit CinB binding to paternal chromosomes to prevent DNA damage from occurring. Another possible mechanism for the rescue effect of CifA is that it disrupts the interaction between CifB and its targets. This idea is supported by the finding that drastic differences in the CidB interactome, including CidB’s interaction with the karyopherins, are observed in the presence of CidA [40]. Nonetheless, structural information on both CifA–CifB protein complexes is needed to get a clearer idea of the possible mechanisms through which CifA rescues embryos from CI.

Another central set of issues revolves around when and where the Cifs cause their CI-inducing modifications. The Cifs might enzymatically modify the sperm nucleus—e.g., by altering paternal chromatin or DNA—and these modifications would then be carried into the egg upon fertilization, resulting in CI [41,102]. CifB has not been shown experimentally to be carried into the fertilized embryo, so it is conceivable that it might only modify sperm at pre-fertilization stages, as emphasized in the HM model. CifA supplied by the infected egg could then rescue the defect by reversing or neutralizing such modifications in the paternal pronucleus, as proposed in part of the HM model [102]. In the TA model, however, CifB could similarly modify sperm precursors during spermatogenesis and sustain this modification by being carried into the embryo. By binding CifB, CifA supplied in the infected egg will neutralize its activity and allow the repair of the paternal pronucleus [38]. However, it remains puzzling that the paternal CifA rescue factor packaged in the sperm or present in its precursors does not revert or prevent the modification, yet the maternally supplied CifA does. It has been proposed that differences in post-translational modification or cellular environment in sperm and egg may contribute to the different roles of CifA [35,41,105].

Alternatively, sperm from infected males may act as a Trojan horse to deliver Cifs to the egg where the proteins induce deleterious changes in the paternal pronucleus shortly after fertilization (another scenario of the TA model) [38]. One observation supporting this hypothesis is the detection of CidA in mature sperm stored in the spermathecae of female mosquitoes [34]. Though CidB was not detected in the same study, it is important to realize that only specific protein gel bands were excised for mass spectrometry analysis in this study, and none was within the expected gel migration range of CidB based on its known molecular mass [34,106]. From quantitative RNA analysis, *cidA* is also expressed at substantially higher levels than *cidB* [34,37]. In many known toxin–antitoxin systems in free-living bacteria, the antitoxin (antidote) proteins are more rapidly degraded than are the toxin proteins [79]. For CI, CifA may get degraded upon (or right before) egg fertilization, releasing active CifB to modify its targets and induce CI. Interestingly, in early attempts to express recombinant CidA in *E. coli*, the protein was cleaved by the resident *E. coli* Lon protease [24], a member of a family of proteases that degrades multiple proteins including the antitoxins of some *E. coli* TA systems [107,108]. It is possible that the antidote CidA is degraded by insect orthologs of Lon protease or other proteases with the appropriate substrate specificity during the early stages of zygote formation and development.

## 8. Future Prospects

Most current *Wolbachia*-related research concentrates on either the evolutionary ecology of *Wolbachia* or the application of *Wolbachia* to vector-borne disease control [109,110]. Relatively few studies have focused on unraveling the biochemical mechanisms of CI or the molecular functions of CI factors. Several reports, however, provide clues about what host pathways might be affected by or mediate *Wolbachia*-induced CI. As noted earlier, analysis of the transcriptional changes in spider mite embryos resulting from incompatible crosses has highlighted the up-regulation of DNA-damage response and chromatin-related genes, among other targets [73].

An intriguing study has implicated host immunity-related genes in CI, most notably a strong increase in expression of the *kenny* gene in the testes of *w*Mel-infected *D. melanogaster* [111]. *Kenny* encodes the fly NEMO/IKKγ protein, a regulatory subunit of the IκB kinase (IKK) complex that controls the NF-κB transcription factor. High levels of *kenny* in the testes lead to reduced embryo hatch rates, and *w*Mel-infected females are able to rescue the defect. Increased apoptosis and reactive oxygen species in the testes were also reported. Interestingly, the mammalian NEMO protein is known to recognize K63-linked polyubiquitin chains and to be ubiquitylated itself with the same type of chains. These activities are important for the function of the NF-κB pathway [112]. Given that CidB is a deubiquitylase with K63 chain preference, there might be a close link between *kenny* and CidB function. A separate report from the same group found that *w*Mel infection also alters the expression of specific microRNAs in the testes, and increased levels of one of them inhibits the expression of *pipsqueak* [113]. *Pipsqueak* encodes a transcriptional regulator, and males with reduced *pipsqueak* levels in the testes cause a CI-like phenotype when mated to uninfected females; *w*Mel infection of females suppresses these defects. It will be interesting to determine if transgenic expression of Cifs in the male germline leads to similar increases in *kenny* or microRNA-mediated decreases in *pipsqueak* in the testes.

It is noteworthy that DUB and nuclease domains similar to those in the *Wolbachia* CidB and CinB proteins have been found in a range of other intracellular bacteria involved in reproductive manipulations. For example, *Rickettsia felis*, a relative of *Wolbachia* known to induce parthenogenesis in the booklouse, was found to carry a plasmid encoding a CifA-CifB-related toxin–antitoxin module in which the toxin contains a pair of PD-(D/E)xK domains and a Ulp-like DUB domain—as well as several other elements [49]. A *Spiroplasma poulsonii* protein, named Spaid, recently shown to be involved in male killing in *Drosophila* species, also contains a DUB domain but from the OTU sequence class [114]. Likewise, a PD-(D/E)xK nuclease domain similar to the ones in CinB has been identified in *Medea*, a selfish genetic element that can induce both maternal lethality and zygotic rescue in the flour beetle *Tribolium castaneum* [115]. Thus, more in-depth biochemical and molecular characterizations of these DUB and nuclease domain-containing factors would help us better understand not only the molecular mechanism behind *Wolbachia*-induced CI but also mechanisms of other forms of reproductive parasitism.

Here we have reviewed our nascent understanding of the biochemistry and mechanistic implications of the recently discovered CI factors in *Wolbachia*. Although we now know they play important roles in CI induction and rescue, we are still in the very early stages of determining the biochemical and cell biological mechanisms of CI. We know even less about how CI is induced by other intracellular bacteria such as *Cardinium* where not even the relevant factors have been identified [26]. Nor do we know the mechanisms of *Wolbachia*-induced male killing, parthenogenesis, or feminization, which are probably caused by distinct factors. Although a TA model for CI (Figure 3) is plausible and fully consistent with available data, other mechanisms certainly have not been ruled out. Identifying the host targets of the Cif enzymes and how they are altered will be an important goal for helping clarify the mechanisms of *Wolbachia*-induced CI. We can anticipate an exciting period of discovery and insight as these decades-old mysteries of endosymbiont-mediated reproductive manipulation are finally unraveled.

## Figures and Tables

**Table 1:** Summary of proposed models for cytoplasmic incompatibility and rescue.

	Model	Mode of CI	Mode of Rescue	Reference
Early models (pre-Cifs)	Mis-timing (“Slow-motion”)	*Wolbachia* infection slows down male pronuclear development. The resultant asynchrony of the parental and maternal pronucleus development causes CI.	Maternal pronucleus in *Wolbachia*-infected egg experiences a similar delay, or *Wolbachia*-infected egg accelerates male pronuclear development, resulting in developmental synchronization with the delayed male pronucleus.	[14,31,32,95,103]
	Titration–Restitution (“Sink”)	A key host component involved in proper chromosome condensation and segregation is removed from male chromosomes by a *Wolbachia* effector.	*Wolbachia*-infected egg replenishes such components to the male chromosomes.	[23,95,98]
	“Lock and Key”	During spermatogenesis, *Wolbachia* deposit a “lock” factor that binds to certain component(s) of the paternal nucleus, interrupting its normal function.	*Wolbachia*-infected egg produces a “key” that specifically interacts with the “lock” and removes it from the paternal material.	[23,99,100,101]
Models proposed after the discovery of Cifs	Toxin-antidote	*Wolbachia* secrete both CI toxin and antidote (CifB and CifA) during spermatogenesis. The rapid degradation of antidote protein in the mature sperm or fertilized egg activates the toxin which causes chromosomal defects in the male pronucleus.	Antidote protein (CifA) provided by *Wolbachia*-infected egg rescues the defect caused by the toxin, likely through direct binding to the toxin.	[24,38,100]
	Two-by-one	A pair of *Wolbachia* effectors CifA and CifB function together to cause CI. CifA and CifB can induce CI either in a “toxin-antidote” mode, similar to described above; or in a “host modification” (HM) mode, where they induce CI by delaying the paternal pronucleus or modifying testis-specific host factor(s).	CifA protein produced in the infected egg rescues CI by acting as an antidote or modifying certain host factors in the embryo.	[41]
